# Effects of coach-delivered verbal encouragement on the physiological and psychological responses of adolescent players in small-sided basketball games

**DOI:** 10.3389/fpsyg.2024.1392668

**Published:** 2024-05-15

**Authors:** Ala Khayati, Faten Sahli, Hatem Ghouili, Rabeh Labbadi, Okba Selmi, Hajer Sahli, Nidhal Jebabli, Amir Romdhani, Makram Zghibi, Monoem Haddad

**Affiliations:** ^1^Higher Institute of Sport and Physical Education of Ksar Said, University of Manouba, Manouba, Tunisia; ^2^High Institute of Sports and Physical Education of Kef, University of Jendouba, El Kef, Tunisia; ^3^Physical Activity, Sport and Health, National Observatory of Sports, Tunis, Tunisia; ^4^Physical Education Department, College of Education, Qatar University, Doha, Qatar

**Keywords:** athletic performance, coaching strategies, RPE, profile of mood state, physical activity enjoyment

## Abstract

**Introduction:**

The confluence of physiological and psychological dynamics is fundamental to athletic performance, particularly in basketball, where physical skill and mental resilience are imperative. While the role of verbal encouragement (VE) as a catalyst for enhancing performance has been explored in various sports disciplines, its specific effects within the basketball have not been adequately examined. Addressing this gap, the current study zeroes in on the influence of coach-delivered VE on the physiological and psychological responses of adolescent basketball players engaged in small-sided games (SSG), providing a focused analysis of how directed encouragement can modulate performance and experience in young athletes. This study aimed to investigate the effects of coach-delivered verbal encouragement on the psychological and physiological responses of adolescent basketball players.

**Methods:**

Sixteen male participants (age: 16.93 ± 0.36 years; height: 176.8 ± 0.8 cm; body mass: 73.43 ± 12.57 kg; BMI: 21.70 ± 3.55) were allocated to a Verbal Encouragement Group (VEG, *n* = 8, mean age: 16.80 ± 0.44) and a Control Group (CG, *n* = 8, mean age: 17.06 ± 0.26). Each participant engaged in four sessions of small-sided games (SSGs) consisting of four players per side in a 14 × 10 m pitch.

**Results:**

The findings revealed significant benefits of coach-delivered verbal encouragement on both the physical and psychophysiological responses of the players (*p* < 0.05), including increased physical enjoyment, positive mood state, lower heart rate, and higher physical activity intensity level.

**Discussion:**

Coaches should incorporate verbal encouragement strategies during SSGs to enhance player performance and optimize both psychological and physiological adaptations.

## Introduction

Basketball players require robust physiological capabilities, such as endurance, strength, agility, and motor skills, to perform the physically demanding actions of the game, from sprinting and jumping to precise ball-handling (Maggioni et al., 2019; Zhang et al., 2019; Papla et al., 2022; Kumari et al., 2023). Equally important are psychological strengths, including mental skills and mental toughness, which enable them to make tactical decisions, communicate effectively, and remain resilient under the high-pressure conditions of competitive play. Previous research highlighted that interpersonal aspects in sports may include communication, and collaboration with coaches, teammates (Bowles and O’Dwyer, 2022; Nunes et al., 2022).

Body of evidence supports the effectiveness of small-sided games (SSGs) as a meth-od for simultaneously enhancement of physical and physiological capacity, as well as technical and tactical skills of basketball players (Matthew and Anne, 2009). These games are recognized as a highly effective strategy for enhancing the endurance of basketball players, given the sig-nificant correlation between aerobic power and the ability to consistently exert high-intensity efforts during such games (Aguiar et al., 2015; Selmi et al., 2017, 2018; Bujalance-Moreno et al., 2019). Additionally, employing positive verbal encouragement in communication contributes to cultivating a supportive team environment, thereby enhancing collaboration, cohesion, and overall team performance.” Considering their capacity to simultaneously promote the development of muscular, physiological, and technical-tactical qualities essential for competitiveness, prior studies have suggested that SSGs emerge as a notably valuable training technique in basketball (Clemente et al., 2020). Utilizing positive verbal encouragement in communication plays a pivotal role in fostering a supportive team environment, thereby reinforcing collaboration, cohesion and overall team performance (Sahli et al., 2023).

Further research has consistently shown that VE has a positive impact on the psychological state of athletes. This effect enhances their selfconfidence, motivation and con-centration during training and competition (Selmi et al., 2017; Aydi et al., 2022; Sahli et al., 2023).

An expanding body of research contends that verbal encouragement from coaches can enhance motivation and physical performance across various contexts and activities, including Small-Sided Games (SSGs) (Rampinini et al., 2007; Selmi et al., 2017; Sarmento et al., 2018). It is noteworthy that the verbal guidance provided by a coach can exert a notable influence on team performance (Cushion and Jones, 2001), making coach speech a subject of prior research focus (Lorenzo et al., 2015). Intriguingly, athletic performance has demonstrated a significant improvement under verbal encouragement not only during SSGs but also in competitive settings (Mazzetti et al., 2000).

An expanding body of research contends that VE from coaches can enhance motivation and physical performance across various contexts and activities, including SSGs (Rampinini et al., 2007; Selmi et al., 2017; Sarmento et al., 2018). It is noteworthy that the verbal guidance provided by a coach can exert a notable influence on team performance (Cushion and Jones, 2001). Therefore, the coach speech has been the focus of earlier research (Lorenzo et al., 2015). Interestingly, athletic performance has demonstrated a significant improvement under VE not only during SSGs but also in competitive settings (Mazzetti et al., 2000).

The compelling evidence supporting the efficacy of VE during SSGs, along with the important role motivation plays in trainings (Hidi and Harackiewicz, 2000) are major catalysts for this research. The present study, aimed to examined the impact of coach VE during SSGs on physiological and affective aspects of adolescent basketball players during trainings or competition.

This study is motivated by the imperative to explore the effects of Experiential Learning (EV) within the niche of sport science education. While existing research has delved into the influence of EV on professional athletes spanning different age groups, as well as on physical education students at earlier educational stages like elementary school, there exists a dearth of comprehension regarding its impact on sport science students. We posit that the consistent and iterative provision of EV by the instructor will result in noteworthy enhancements in performance, perceived effort, and psychological responses among sport science students.

## Materials and methods

### Ethical approval

The study was conducted in accordance with the Declaration of Helsinki, and the protocol was fully approved by the research ethics committee of the High Institute of Sports and Physical Education of Kef. Additionally, this research adheres to ethical guidelines for research practices in sports medicine and exercise science (Guelmemi et al., 2024).

### Participants and study design

Before recruitment, we used the free software GPower version 3.1.9.6 (Germany) for determining the optimal sample size. Thus, a power analysis for a repeated measures ANOVA was conducted, focusing on a within-between interaction. The analysis was predicated on an anticipated effect size (f) of 0.4, indicative of a moderately large effect, with an alpha error probability set at 0.05 and a desired statistical power of 0.8. The experimental design comprised two groups, each subjected to two measurements, with an assumed correlation of 0.5 between these measurements. No correction for sphericity (*ε* = 1) was applied.

The power analysis yielded a noncentrality parameter (λ) of 10.24. Additionally, the critical *F*-value was calculated to be 4.60, serving as the F-distribution’s cut-off point for null hypothesis rejection. To reach the predetermined power, a sample size of 16 was deemed necessary, as reflected by the actual power computation of 0.85, marginally surpassing our target. This sample size is considered sufficient to detect the expected effect size with the specified power.

In this randomized controlled design, 16 male basketball players (age: 16.93 ± 0.36 years; height: 176.8 ± 0.8 cm; body mass: 73.43 ± 12.57 kg; BMI: 21.70 ± 3.55) were randomly assigned to two groups (eight players per group). Players were first stratified based on a baseline assessment of their technical abilities, including dribbling speed, shooting accuracy, and defensive skills. Players were then randomly assigned to either the Verbal Encouragement Group (VEG) or the Control Group (CG) within each skill tier using a computer-generated random number sequence to ensure balanced skill levels. The control group (SSGNE) and the experimental group (SSGE) were constituted accordingly. These participants were recruited from the Ain Drahm basketball team. Throughout the study, participants engaged in three training sessions per week, with each player required to partake in four sessions of small-sided games (SSG). The SSG sessions were conducted on half of a basketball court measuring 14 × 15 m, accommodating two 4v4 SSGs, with the same players consistently involved. Notably, unlike the control group (SSGNE), the SSGE group received verbal encouragement from the coach, both defensively and offensively. Among the verbal encouragement group, the physical education teacher encourages the students using specific instruction related to team sports (Andreacci et al., 2002). Using a cheer every 20 s, the teacher became accustomed to the Protocol (Sánchez-Sánchez et al., 2017). All participants had no reported injuries or illnesses before or during the study. Participants and their parents voluntarily agreed to participate in the study and gave written informed consent after a detailed explanation about the aims and risks involved in the research.

### Procedures

This study was conducted during the 2022–2023 mid-season. Prior to SSGs, anthropometric characteristics were collected (Table 1). Before beginning the experimental testing, anthropometric measurements were taken.

**Table 1:** Demographic and anthropometric characteristics of participants (mean +/− standard deviation) (*n* = 16).

	Age (year)	size (cm)	weight (kg)	Waist size	BMI
Mean	15.92	1.84	74.42	83.71	21.70
Standard deviation	0.35	0.06	12.56	10.60	3.55

BMI, body mass index.

All participants underwent a standardized 15-min warm-up comprising low-intensity running, coordination movements, and dynamic stretching, including high knee lifts, butt kicks, straight-line skipping, etc., followed by a series of 3 × 10 m sprints. 3 min of recovery separated the warm-up from the beginning of the SSG.

The 4 experimental sessions comprised 4-a-side and were conducted on distinct days, each with a one-week interval between sessions. All experimental sessions took place on the same indoor basketball room. The pitch measuring 14 × 15 m and at a consistent time of day (between 5:00 and 6:00 PM) to mitigate the impact of circadian variations. The training consisted of three SSG sets of 4 min duration each, with 90 s of passive rest between sets. Four training sessions were conducted on different days (twice a week), during each players took part in three sets of SSGs. During each experimental session, participants were divided into two groups, with 8 students (4 vs. 4) per-forming SSGs on the half basketball field under teacher verbal encouragement, while the other eight performed SSGs with no verbal encouragement on the other half of the court. HR was monitored throughout each training session. RPE was recorded, and the PACES was completed 5 min after the end of each SSG. The profile of mood state (POMS) recorded before and after each training session. Players were familiarized with the RPE scale, PACES scale, POMS questionnaire and the Small-Sided Game (SSG) regimen before the study commenced. Data for each SSG session were consistently collected by the same group of researchers.

### Small-sided games

The two groups participated in a 4vs4 SSG on a half basketball field, with (SSGE) or without teacher verbal encouragement (SSGNE) (Table 2). The duration of each Small-Sided Game (SSG) was precisely regulated, consisting of 3 bouts lasting 4 min each, with 90 s of passive recovery between bouts. The players were instructed to exert maximum effort during the games while aiming to maintain possession of the ball for the longest possible duration.

**Table 2:** Small-sided games practiced by the students.

Variables	Teachers’ encouragement						Format	Field size
	with encouragement			without encouragement				
	before	during	after	before	During	After		
POMS	×		×	×		×	4vs.4	14 × 15 m
PACES			×			×		
RPE			×			×		
FC		×			×			

Each bout commenced with the ball in the air. In the SSGE condition, two coaches strategically positioned around the pitch facilitated game continuity by providing new balls when necessary. Importantly, they actively encouraged players using team sports related terminology such as “Go,” “Good job,” “Excellent,” “Come on, push it,” and “Keep it up,” “Let us go!” “Stay focused and keep pushing!” “Keep your head in the game!” “Let us see some hustle out there!” “Great shot, keep firing!” “Nice move!” “Defense! Lock it down!” “Great maneuver; repeat that” (Lorenzo et al., 2015). The week before data collection, the coach was familiarized with the verbal encouragement procedure. Sánchez-Sánchez et al. (2017) device was used to continuously track the verbal encouragement level each 20 s.

### Load of the small-sided games

Following training in device utilization, player’s heart rate was measured throughout the various SSGs using individual heart rate monitors (Polar Team Sport System, Po-lar-Electro OY, Kempele, Finland). The highest heart rate recorded during each session was the maximum heart rate (HR max). PER was measured using the 10-point scale proposed by Foster et al. (2001), 1 min after each set corresponding to each SSG format. The scale was thoroughly explained to the participants. The question “How did it go and how did you feel about the exercise? was addressed to each player individually in the absence of peers, all while being blinded to the points awarded to each of them (Table 3).

**Table 3:** Results of two-factor (ANOVA): games factor (SSGE and SSGNE), session’s factor and their interaction on PACES, RPE, and HRmax.

Variables	Sessions			Game			Interaction		
	*F*	Sig	E.S	*F*	Sig	E.S	*F*	Sig	E.S
Paces	3.717*	0.022	0.271	0.123	0.733	0.012	1.681	0.192	0.144
RPE	4.770**	0.008	0.323	1.966	0.191	0.164	9.724***	0.000	0.493
HRmax	0.512	0.677	0.049	5.233*	0.045	0.344	0.512	0.677	0.049

### Enjoyment of physical activity

The assessment of players’ enjoyment of physical activity (PACES) utilized an 18-item scale gaging the perceived benefits of engaging in physical activity (Sánchez-Sánchez et al., 2017). Partici-pants employed a 7-point bipolar rating scale, ranging from 1 (It’s very enjoyable) to 7 (It’s not fun at all), to express ‘how they feel right now about the physical activity they did.’ Note that the scores for 11 items were reversed. The cumulative scores of all items were then summed to derive the overall physical activity enjoyment score, spanning from 18 to 126. Higher PACES scores are indicative of elevated enjoyment levels. Each student received an individual paper copy of the scale (Hopkins et al., 2009).

### Profile of the mood-state

The Profile of Mood States (POMS) questionnaire, consisting of 65 items, was used to evaluate six different components of mood states: tension-anxiety, depression-depression, hostility-anger, vigor-activity, fatigue-inertia, and confusion-perplexity. Each item was rated on a 5-point Likert scale, where 0 represented “Not at all” and 4 represented “Extremely.” The total mood disturbance score (TMD), which is the sum of the T-scores for each of the six POMS subscales, can be calculated by adding the T-scores for the five sub-scales measuring negative mood, deducting the *T*-score measuring positive mood, and adding a constant of 100 to prevent negative numbers [TMD = ((Anger + Confusion + Depression + Fatigue + Tension) - Vigor) + 100]. Participants responded to the POMS questionnaire individually.

### Statistical procedures

Data is presented as Mean (M) and standard deviation (SD). The Kolmogorov–Smirnov test was used to verify the normality assumption before using parametric tests. The effects of play (SSGE and SSGNE), time (pre- and post-training session), and their interaction on the POMS score were examined using a two-factor analysis of variance (ANOVA). (2) The interaction between “sessions,” “the effect of play” (SSGE and SSGNE), and “PACES, RPE, and HRmax.” To compare the physiological and psychological out-comes following the two game modalities (with and without encouragement), the independent samples *t*-test was used. A rigorous assessment of the differences between SSGE and SSGNE was conducted using the magnitude of change expressed by Cohen’s d coefficient (Cohen, 1992). According to Hopkins et al. (2009), magnitude scales with values between 0 and 0.20, >0.20 to 0.50, >0.50 to 0.80, and > 0.80 were categorized as trivial, small, medi-um-sized, and large, respectively. The significance level was set at *p* < 0.05, and analyses were carried out using the Statistical Package for the Social Sciences (v26.0, SPSS, SPSS Inc., Chicago, IL, USA).

## Results

### Physiological responses

A significant effect of the sessions factor (*p* = 0.02, ES = 0.27) on PACES scores (Table 3).

The results presented in Table 3 show significant effects of the session factor (*p* = 0.008, ES = 0.34) and interaction factor (sessions * games) (*p* = 0.045, ES = 0.14) on the RPE scores. Concerning the HR max, there is a significant effect only on the games factor (*p* = 0.045, ES = 0.34) (Table 3).

The results illustrated in Table 4 showed significant differences between SSGE and SSGNE for the variables HRmax in sessions S2 and S4 (*p* = 0.025, ES = 0.60; *p* = 0.017, ES = 0.63), and for the RPE score in session S4 (*p* = 0.032, ES = 0.58).

**Table 4:** Comparison of PACES, RPE, and HR max between small-sided games with encouragement (SSGE) and small-sided games without encouragement (SSGNE).

Variables		With ENC.		Without ENC.		*T*	*p*	E.S
		*M*	SD	*M*	SD			
Paces	S1	5.84	1.84	5.00	2.00	0.752	0.469	0.21
	S2	4.67	1.22	5.17	1.73	−0.582	0.574	0.16
	S3	4.17	1.48	4.84	1.17	−0.869	0.405	0.24
	S4	3.84	1.17	4.50	1.05	−1.040	0.323	0.28
RPE	S1	76.84	6.86	76.50	6.54	0.086	0.933	0.02
	S2	81.84	6.18	77.17	7.99	1.132	0.284	0.31
	S3	83.67	6.25	76.17	9.54	1.611	0.138	0.42
	S4	84.00	6.32	74.00	7.50	2.495	0.032	0.58
HRmax	S1	188.00	11.15	183.00	11.55	0.762	0.463	0.21
	S2	192.33	5.17	182.00	8.07	2.641	0.025	0.60
	S3	191.00	7.09	181.67	10.70	1.779	0.106	0.45
	S4	190.83	6.62	180.17	6.34	2.852	0.017	0.63

M, mean; SD, standard deviation; ES, effect size.

### Physical enjoyment

The analysis of PACES scores across small-sided games, with encouragement (SSGE) and without encouragement (SSGNEE), revealed no statistically significant differences (Table 4).

### Mood state

We found a significant main effect of Time on the 6 mood states tension (S4: *p* = 0.001), confusion (S3: *p* = 0.05), anger (S1: *p* = 0.05, S2: *p* = 0.048), depression (S1: *p* = 0.028, S3: *p* = 0.045, S4: *p* = 0.042), fatigue (S1: *p* = 0.001, S2: *p* = 0. 003 and S4: *p* = 0.034), Vigor (S2: *p* = 0.02, S3: *p* = 0.005 and S4: *p* = 0.04) and on TMD (S2; *p* = 0.027, S3; *p* = 0.002 and S4; *p* = 0.038), as well as a significant effect of the interaction (time * games) on TDG in session S3 (*p* = 0.009, ES = 0.508) (Table 5).

**Table 5:** Results of two-factor ANOVA: “Games” factor (SSGE and SSGNE), “Time” factor (pre- and post-training session) and their interaction on the POMS score.

Variables		Time			Game			Interaction		
		*F*	SIG	E.S	*F*	SIG.	E.S	*F*	SIG	E.S
Tension	S1	4.71	0.055	0.320	10.001	0.970	0.000	10.18	0.304	0.105
	S2	13.85	0.004	0.581	0.054	0.823	0.005	10.54	0.243	0.133
	S3	9.15	0.013	0.478	0.00	1.000	0.000	20.29	0.162	0.186
	S4	49.00	0.000	0.831	0.08	0.784	0.008	10.00	0.341	0.091
Confusion	S1	6.64	0.028	0.399	0.002	0.962	0.000	0.09	0.780	0.008
	S2	3.74	0.082	0.272	0.05	0.837	0.004	0.60	0.458	0.056
	S3	9.56	0.011	0.489	0.01	0.929	0.001	0.15	0.707	0.015
	S4	0.42	0.5	0.040	0.15	0.709	0.015	00.05	0.835	0.005
Anger	S1	14.31	0.004	0.589	0.61	0.453	0.057	0.050	0.828	0.005
	S2	5.07	0.048	0.336	0.39	0.547	0.037	30.53	0.090	0.260
	S3	9.31	0.012	0.482	0.05	0.827	0.005	0.08	0.787	0.008
	S4	4.50	0.060	0.310	0.56	0.475	0.052	0.50	0.496	0.048
Depression	S1	15.21	0.003	0.603	0.005	0.946	0.000	1.7	0.223	0.195
	S2	13.26	0.005	0.570	0.22	0.655	0.021	0.42	0.535	0.040
	S3	10.91	0.008	0.522	0.84	0.381	0.077	2.73	0.130	0.214
	S4	3.92	0.076	0.281	0.50	0.497	0.047	1.74	0.217	0.148
Fatigue	S1	15.42	0.003	0.036	0.26	0.625	0.025	0.07	0.799	0.007
	S2	66.82	0.000	0.870	1.43	0.260	0.125	1.37	0.270	0.120
	S3	11.40	0.007	0.533	4.44	0.062	0.307	0.93	0.358	0.085
	S4	16.85	0.002	0.628	0.93	0.357	0.085	0.29	0.604	0.028
Vigor	S1	0.38	0.553	0.226	0.160	0.697	0.016	0.17	0.691	0.016
	S2	13.18	0.005	0.569	0.29	0.606	0.028	4.42	0.062	0.306
	S3	22.24	0.001	0.690	0.07	0.807	0.006	9.31	0.12	0.482
	S4	8.46	0.16	0.458	0.45	0.518	0.043	4.55	0.059	0.313
TMD	S1	2.92	0.118	0.226	0.146	0.710	0.014	0.02	0.917	0.001
	S2	13.33	0.004	0.571	0.11	0.747	0.011	2.45	0.149	0.197
	S3	38.58	0.000	0.794	0.42	0.532	0.040	10.33	0.009	0.508
	S4	6.60	0.028	0.397	0.83	0.385	0.076	2.62	0.137	0.207

The comparison of the different mood states before and after SSG showed a significant difference of the 6 mood states at SSGE: Tension (S4; *p* = 0.001), Confusion (S3: *p* = 0.001), Anger (S1: *p* = 0.03, S2: *p* = 0.048), Depression (S1: *p* = 0.028, S3: *p* = 0. 045 and S4: *p* = 0.042), Fatigue (S1: *p* = 0.001, S2: *p* = 0.003 and S4: *p* = 0.034), Vigor (S2: *p* = 0.022, S3: *p* = 0.005 and S4: *p* = 0. 041) as well as the SSGNE: tension (S4: *p* = 0.012), anger (S3: *p* = 0.041), depression (S2: *p* = 0.013), Fatigue (S2: *p* = 0.001, S3: *p* = 0.001and S4: *p* = 0.003) (Tables 6, 7).

**Table 6:** Comparison of different mood states before and after small-sided games with encouragement (SSGE).

Variables		Before		After		*T*	*p*	E.S
		*M*	SD	*M*	SD			
Tension	S1	11.33	6.80	10.33	6.77	2.236	0.076	0.07
	S2	10.33	6.08	8.50	6.41	4.00	0.10	0.06
	S3	12.33	5.42	10.33	5.75	2.449	0.058	0.17
	S4	11.00	6.69	9.66	6.37	6.325	0.001	0.10
Confusion	S1	8.50	3.95	6.67	2.74	2.076	0.093	0.14
	S2	8.33	3.50	6.83	3.25	0.696	0.518	0.08
	S3	8.33	3.50	6.83	3.31	6.708	0.001	0.21
	S4	8.16	2.56	8.00	3.03	0.415	0.695	0.02
Anger	S1	16.50	9.44	15.00	8.74	3.00	0.030	0.08
	S2	15.00	5.40	13.16	4.16	2.607	0.048	0.18
	S3	12.66	2.80	11.83	3.48	1.746	0.141	0.13
	S4	11.16	2.92	9.83	2.13	1.865	0.121	0.25
Depression	S1	11.00	5.66	8.67	4.23	3.070	0.028	0.22
	S2	10.33	4.36	8.66	3.50	2.331	0.067	0.20
	S3	9.16	3.54	7.66	2.33	2.666	0.045	0.24
	S4	9.16	2.56	8.33	2.33	2.712	0.042	0.16
Fatigue	S1	6.84	1.17	8.00	1.27	−7.000	0.001	0.42
	S2	6.50	1.22	8.50	1.04	−5.477	0.003	0.66
	S3	6.66	1.03	7.50	1.04	−1.536	0.185	0.37
	S4	8.16	0.75	10.33	1.75	−2.892	0.034	0.62
Vigor	S1	20.33	5.89	20.50	6.30	−0.107	0.919	0.01
	S2	22.33	4.88	24.83	5.49	−3.273	0.022	0.23
	S3	22.33	4.92	24.66	4.80	−4.719	0.005	0.23
	S4	23.33	5.75	25.50	5.54	−2.735	0.041	0.18
TMD	S1	128.84	19.78	126.00	14.00	0.996	0.365	0.08
	S2	124.66	14.81	117.83	12.18	3.091	0.027	0.21
	S3	120.83	10.53	113.50	10.96	6.102	0.002	0.32
	S4	118.33	8.50	115.66	7.14	2.803	0.038	0.22

M, mean; SD, standard deviation; ES, effect size.

**Table 7:** Comparison of different mood states before and after small-sided games without encouragement (SSGNE).

Variables		Before		After		*T*	*p*	ES
		*M*	SD	*M*	SD			
Tension	S1	11.16	8.47	10.83	8.28	0.791	0.465	0.01
	S2	11.50	8.78	10.83	8.56	1.581	0.175	0.03
	S3	10.66	8.06	9.00	8.40	2.000	0.102	0.04
	S4	12.00	8.00	10.00	8.48	3.873	0.012	0.06
Confusion	S1	8.33	3.38	6.66	3.80	1.581	0.175	0.10
	S2	8.33	3.20	7.16	2.22	2.445	0.058	0.20
	S3	8.33	3.50	7.16	2.13	1.400	0.220	0.19
	S4	8.83	2.63	9.50	2.58	0.500	0.638	0.06
Anger	S1	13.00	6.16	11.66	5.31	2.390	0.062	0.11
	S2	12.50	4.67	12.33	4.45	0.307	0.771	0.01
	S3	13.33	5.92	12.33	5.24	2.739	0.041	0.08
	S4	12.33	4.76	11.66	3.98	1.085	0.328	0.07
Depression	S1	10.16	7.83	9.00	6.72	2.445	0.058	0.07
	S2	11.33	5.39	10.16	5.45	3.796	0.013	0.10
	S3	10.33	3.38	9.83	3.37	2.236	0.076	0.07
	S4	9.83	2.48	9.66	2.58	0.415	0.695	0.03
Fatigue	S1	7.59	3.61	8.83	3.43	−2.169	0.082	0.17
	S2	8.83	4.07	10.33	4.22	−6.708	0.001	0.17
	S3	9.33	3.50	10.83	3.37	−3.503	0.017	0.21
	S4	10.83	3.31	11.50	3.72	−2.988	0.031	0.14
Vigor	S1	21.00	2.09	21.83	2.04	−1.746	0.141	0.19
	S2	22.00	2.60	22.66	2.73	−1.581	0.175	0.12
	S3	22.66	2.73	23.166	3.31	−1.464	0.203	0.09
	S4	22.33	4.41	22.66	4.13	−1.000	0.363	0.03
TMD	S1	124.16	23.59	121.66	23.38	1.946	0.109	0.05
	S2	125.50	23.20	123.16	22.53	1.941	0.110	0.05
	S3	124.33	21.24	122.00	18.89	2.360	0.065	0.05
	S4	126.50	20.24	124.66	17.71	0.714	0.507	0.02

M, mean; SD, standard deviation; ES, effect size.

## Discussion

The present study examined assessed the effect of the sports teacher’s verbal encouragement on the physiological responses, mood state, physical enjoyment and technical-tactical skills of adolescent basketball players during SSGs. The main findings of the present study are that (1) SSGE significantly heightened physiological responses compared to SSGNE, and (2) participants reported a notably improved mood state following SSGE in contrast to SSGNE.

The current investigation demonstrates that verbal encouragement from a sports teacher has a beneficial effect on the physiological responses observed during Small-Sided Games (SSGs). These results support earlier research suggesting that coaches might improve players’ physical and psychological-physiological reactions during basketball drib-bling drills by using verbal cues (Garcia-Rubio et al., 2014). Our results unequivocally show that players can be-come more motivated after verbal support from teachers, and that higher motivation can lead to increased physical effort and the ability to maintain a high work rate during small-sided games. This pattern of outcomes is also consistent with what Rampinini et al. (2007) reported. Rampinini et al. (2007) examined the effects of verbal encouragement on different physiological responses in a number of small-sided game formats on small, medium, and large fields, including 3 vs. 3, 4 vs. 4, 5 against. 5, and 6 vs. 6. Researchers Rampinini et al. (2007) looked into how different physiological reactions were affected by verbal encouragement. They showed that HR and RPE values were significantly higher during gaming sessions when verbal encouragement was provided. Similar beneficial effects were observed when Selmi et al. (2017) examined the physiological reactions of young soccer players to verbal encouragement from coaches during 4 vs. 4 SSG.

Coach verbal encouragement has also been shown to have a positive impact during basketball games (Garcia-Rubio et al., 2014). Furthermore, Edwards et al. (2018) reported that verbal encouragement improved performance and motivation during endurance activities, resulting in significant implications for health, adherence, and physical performance when using a hands-on intervention. These findings may lead to the conclusion that motivation, which can be positively influenced by teacher or coach verbal encouragement, can affect the physiological responses and internal intensity arising during SSGs.

The results of the current investigation showed that there was no discernible difference in the physical enjoyment of activity between SSGE and SSGNE. Nonetheless, PACES scores were significantly impacted by the session component. Our results conflict with those of Kilit et al. (2019), who hypothesized that verbal encouragement positively affects participants’ physical satisfaction and commitment to exercise. Numerous studies have shown that physical enjoyment plays a role in the favorable feelings associated with physical activity (Selmi et al., 2020). The PACES scale has been shown to be useful in assessing athletes’ experiences of physical pleasure in a number of studies (Selmi et al., 2020). As Selmi et al. (2017) demonstrated, providing encouragement during training activities can increase athletes’ motivation and good actions. One of the primary elements influencing the desire to engage in physical activity.

The findings of the present study demonstrated a significant positive impact of coach verbal encouragement on mood state during SSGs. In line with our findings, Selmi et al. (2020) found that during integrated training, players generally reported significant improvements in their mood state following exposure to coach verbal encouragement. The POMS is frequently used to assess participants’ psychological responses during physical activity and training.

In our study small-sided SSGE and SSGNE games lead to a decrease in TMD (tension: S4: *p* = 0.001and ES = 0.17), vigor (S2: *p* = 0.022, ES = 0.23, S3; *p* = 0.005, ES = 0.23, S4; *p* = 0.041, ES = 0.18). This indicates that coach verbal encouragement can trigger positive mood state during basketball SSGs. These findings are in line with those presented by Sparkes et al. (2020), who looked at how SSG affected mood. Increased vigor and decreased tension are typically associated with decreases in TDG following SSGE. We believe that the motivational aspects of SSGs in this study may help explain the decreases observed in TDG. More importantly, coaches’ verbal encouragement combined with basket-ball specific training seems to improve mood state.

Overall, the results about students’ subjective perceptions of exercise and their enjoyment of physical activity point to EV as having a major influence. It appears that students not only put up more effort under EV, but also felt the experience more pleasurable and re-warding, as seen by the combination of higher perceived effort and greater enjoyment. It is significant to highlight that although the data show the benefits of exercise, more research is needed to determine the precise processes underlying these benefits. However, the relationship between coaches and athletes, as well as teachers and students, significantly influences the effectiveness of verbal encouragement (VE). A foundation of trust and rapport ensures that encouragement is received as constructive, fostering a receptive and positive learning environment. Effective communication tailored to individual needs enhances motivational impacts, with consistency and authenticity in encouragement crucial for building confidence and self-esteem. An open feedback loop allows for adjustments in coaching or teaching methods, while cultural sensitivity ensures the relevance of encouragement. Empowering athletes and students promotes autonomy, boosting intrinsic motivation. Over time, sustained positive interactions help solidify these relationships, enabling continuous personal and performance development, illustrating that VE is more than motivational words, it’s a strategic approach to nurturing growth and excellence in sports and education. Moreover, Future studies should examine variables including the frequency and timing of EV, individual variations in how they react to encouragement, and possible interactions between EV and other performance enhancing techniques.

Finally, since the mental skills in sports are always linked to the social and cultural environment (Guelmemi et al., 2015; Eubank et al., 2017; Gill et al., 2017) it would be beneficial to exam the effect of VE in various cultures and contexts.

It is important to consider various constraints when interpreting the current findings. First of all, the tiny study sample was a result of the challenge of finding a lot of subjects who were all the same. Additionally, a single SSG format and a single age cohort of soccer-specialist pupils were used in the assessment. To in-crease the applicability of the current findings, future studies comparing the two SSG conditions should use participants of varying ages and varying levels of SSG intensity deter-mining variables (i.e., different game rules, duration of each bout, pitch size, number of players, and the presence of goalkeepers). Lastly, it would be fascinating to link these reactions to technical time-motion parameter elements (such as distance traveled, number of sprints, high-intensity runs, etc.) since physical.

As practical implications, the study provides several important implications for sports training and coaching:

The findings suggest that verbal encouragement from coaches can significantly enhance players’ performance metrics such as heart rate and perceived exertion levels. This indicates that strategic verbal cues during training can help players maintain higher intensity levels and improve overall game performance.

The study also highlights the psychological benefits of verbal encouragement, showing improved mood states among players receiving encouragement. This suggests that verbal encouragement not only enhances physical performance but also positively impacts athletes’ psychological well-being, which can be crucial for their overall development and performance consistency.

For coaches, the study underscores the importance of incorporating motivational strategies into their coaching routines. Understanding the positive impact of verbal encouragement can help coaches better engage with their athletes, fostering a supportive environment that enhances both skill development and mental resilience.

The implications for training design suggest that integrating verbal encouragement into regular practice sessions can lead to better engagement and performance from athletes. This could influence how training sessions are structured, emphasizing not just the physical but also the motivational aspects of sports training.

Given the observed improvements in mood and cohesion, coaches might also use verbal encouragement to foster better team dynamics and collaboration among players. This could be particularly useful in team sports where cooperation and team spirit are crucial for success.

## Conclusion

The present study explored the impact of coach verbal encouragement on adolescent basketball players’ performance, encompassing physiological markers such as heart rate (HR) and ratings of perceived exertion (RPE), as well as psychological factors like enjoyment of Physical Activity Enjoyment Scale (PACES) activities and mood state. Our findings revealed significant positive effects of verbal encouragement on the RPE scores, mood state (measured by the Total Mood Disturbance index), and technical performance, particularly in passing and running shots. These results emphasize the crucial role of verbal support from coaches and physical education instructors during small-sided matches, not only in enhancing physiological responses but also in nurturing a positive mental attitude among players. To comprehensively understand the external load in such games, future research should consider integrating additional variables such as travel distance, speed zones, and acceleration/deceleration patterns. However, it’s vital to acknowledge the limitations of our study, including the need for a larger sample size to improve generalizability and the caution required when extending findings beyond young basketball players. Moreover, the relatively small sample size may influence the reliability of our results and should be taken into account during interpretation.

## Data availability statement

The raw data supporting the conclusions of this article will be made available by the authors, without undue reservation.

## Ethics statement

The studies involving humans were approved by the ethics committee of the High Institute of Sports and Physical Education of Kef. The studies were conducted in accordance with the local legislation and institutional requirements. Written informed consent for participation in this study was provided by the participants’ legal guardians/next of kin.

## Author contributions

AK: Conceptualization, Data curation, Formal analysis, Funding acquisition, Investigation, Methodology, Project administration, Resources, Software, Supervision, Validation, Visualization, Writing – original draft, Writing – review & editing. FS: Conceptualization, Data curation, Formal analysis, Funding acquisition, Investigation, Methodology, Project administration, Resources, Software, Supervision, Validation, Visualization, Writing – original draft, Writing – review & editing. HG: Data curation, Formal analysis, Methodology, Supervision, Validation, Writing – original draft, Writing – review & editing. RL: Funding acquisition, Methodology, Supervision, Validation, Visualization, Writing – review & editing. OS: Data curation, Formal analysis, Methodology, Supervision, Validation, Visualization, Writing – review & editing. HS: Methodology, Supervision, Validation, Visualization, Writing – original draft, Writing – review & editing. NJ: Supervision, Validation, Visualization, Writing – review & editing. AR: Data curation, Formal analysis, Funding acquisition, Investigation, Methodology, Writing – review & editing. MZ: Project administration, Supervision, Validation, Visualization, Writing – review & editing. MH: Supervision, Validation, Visualization, Writing – review & editing.
